# Long‐term effects of an oral elemental nutritional supplement on post‐gastrectomy body weight loss in gastric cancer patients (KSES002)

**DOI:** 10.1002/ags3.12290

**Published:** 2019-10-10

**Authors:** Yutaka Kimura, Kazuhiro Nishikawa, Kentaro Kishi, Kentaro Inoue, Jin Matsuyama, Yusuke Akamaru, Shigeyuki Tamura, Junji Kawada, Tomono Kawase, Ryohei Kawabata, Yoshiyuki Fujiwara, Hitoshi Kanno, Takeshi Yamada, Toshio Shimokawa, Hiroshi Imamura

**Affiliations:** ^1^ Department of Surgery Faculty of Medicine Kindai University Osaka‐Sayama Japan; ^2^ Department of Surgery National Hospital Organization Osaka National Hospital Osaka Japan; ^3^ Department of Surgery Osaka Police Hospital Osaka Japan; ^4^ Department of Surgery Kansai Medical University Hirakata Japan; ^5^ Department of Gastroenterological Surgery Higashiosaka City Medical Center Higashiosaka Japan; ^6^ Department of Surgery Ikeda City Hospital Ikeda Japan; ^7^ Department of Surgery Yao Municipal Hospital Yao Japan; ^8^ Department of Surgery Kaizuka City Hospital Kaizuka Japan; ^9^ Department of Surgery Toyonaka Municipal Hospital Toyonaka Japan; ^10^ Department of Surgery Osaka Rosai Hospital Sakai Japan; ^11^ Division of Surgical Oncology Department of Surgery Faculty of Medicine Tottori University Tottori Japan; ^12^ Department of Digestive Surgery Nippon Medical School Tokyo Japan; ^13^ Clinical Study Support Center Wakayama Medical University Wakayama Japan

**Keywords:** body weight loss, distal gastrectomy, nutritional intervention, oral elemental diet, total gastrectomy

## Abstract

**Aim:**

The present study aimed to evaluate the efficacy of short‐term nutritional intervention with an oral elemental diet (ED; Elental; EA Pharma Co., Ltd, Tokyo, Japan) at 300 kcal/day for 6‐8 weeks in the early post‐gastrectomy period on postoperative long‐term body weight loss (BWL).

**Methods:**

We analyzed consecutive patients who were randomly divided to receive the regular diet with or without ED. The control group received regular diet alone post‐gastrectomy, whereas the ED group received 300 kcal ED plus regular diet for 6‐8 weeks. Primary endpoint was percentage (%) BWL (body weight loss; body weight before surgery minus that at postoperative 1 year) by surgical type. Secondary endpoints included changes in nutrition‐related blood parameters.

**Results:**

Of the patients in the original trial, 106 were eligible for efficacy analyses. %BWL at postoperative 1 year was significantly lower in the ED group than in the control group among patients who underwent total gastrectomy (TG) (n = 19 and n = 17, respectively; 9.66 ± 5.98% [95% confidence interval, CI: 6.77‐12.54] vs 15.11 ± 6.78% [95% CI: 11.63‐18.60], *P* = .015), but not in patients who underwent distal gastrectomy (n = 38 and n = 32, respectively; 5.81 ± 7.91% [95% CI: 3.21‐8.41] vs 5.96 ± 6.20% [95% CI: 3.72‐8.19], *P* = .933). In multivariate analysis, ED was the only factor affecting %BWL at postoperative 1 year among patients who underwent TG.

**Conclusions:**

Daily nutritional intervention (300 kcal/day ED) for 6‐8 weeks reduced %BWL not only at postoperative 6‐8 weeks but also at 1 year in patients who underwent TG.

## Abstract 2

Daily nutritional intervention (300 kcal/day elemental diet) for 6‐8 weeks reduced %body weight loss not only at postoperative 6‐8 weeks but also at 1 year in patients who underwent total gastrectomy.

## Graphical abstract

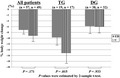

## 1 INTRODUCTION

Gastrectomy for gastric cancer is one of the most common gastroenterological operations in Japan.^1^ After a gastrectomy, patients experience reduced nutrient (energy) intake because of decreased food retention in the stomach and hypofunction of intestinal digestion as a result of upper‐gastrointestinal dysfunction, which result in post‐gastrectomy syndrome and malnutrition.^2^, ^3^, ^4^, ^5^ For these reasons, gastrectomy almost invariably causes body weight loss (BWL). In previous studies, patients who underwent distal gastrectomy (DG) showed BWL of 6%‐10% at 1 year after surgery, whereas those who underwent total gastrectomy (TG) showed BWL of 15%‐18% at postoperative 1 year.^6^, ^7^, ^8^ Weight loss correlates with a decline in postoperative quality of life and worsens the long‐term prognosis of gastric cancer patients.^9^ BWL at 1 month after surgery affects compliance with adjuvant S‐1 chemotherapy and survival of gastric cancer patients.^10^, ^11^ Therefore, to improve the quality of life and prognosis of gastrectomized patients, it is essential to suppress weight loss early after gastrectomy. In a prospective randomized control trial, we have already shown that daily nutritional intervention with an oral elemental diet (ED) at 300 kcal/day for 6‐8 weeks attenuated %BWL within this short period in post‐gastrectomy patients, especially in those who underwent TG.^12^

Conversely, low body mass index (BMI) is generally associated with poor prognosis regardless of disease (cancer or other illnesses). In post‐gastrectomy patients, who tend to experience decreases in BMI, high rates of BMI decline and continuous BMI reduction by 1 year postoperatively are correlated with worse prognosis.^9^ Therefore, suppression of postoperative weight loss in the long term is as important as that during the short period after surgery. However, it is not clear whether short‐term ED in the early postoperative period remains effective in suppressing BWL in the long term. It would be interesting to determine whether the BWL suppression caused by short‐term ED is maintained in the long term in the absence of subsequent planned nutritional intervention. In the present study, we examined the effect of giving short‐term oral ED on postoperative long‐term weight loss in post‐gastrectomy patients according to operation type.

## 2 MATERIALS AND METHODS

### 2.1 Subjects

In this study, we examined patients registered in an original multicenter randomized phase II trial who underwent curative DG or TG for gastric adenocarcinoma at the participating hospitals. Details of the original trial have been reported previously.^12^ The original study was conducted in accordance with the World Medical Association Declaration of Helsinki, and the protocol was approved by the institutional review board of each hospital. Written informed consent was obtained from all patients. This study was conducted in consecutive patients recruited into the original trial, which was registered in the University Hospital Medical Information Network Clinical Trials Registry (UMIN000023455).

The outline of the original trial is described below. Eligibility criteria were as follows: (i) previously untreated (other than gastrectomy) and histopathologically confirmed gastric adenocarcinoma; (ii) age ≥20 years; (iii) clinical stage I, II, or III; (iv) Eastern Cooperative Oncology Group performance status of 0‐2; (v) curative resection at the end of surgery; (vi) ability for oral intake; (vii) provision of written informed consent before randomization; and (viii) absence of any severe postoperative complications between surgery and randomization. Exclusion criteria were as follows: (i) diagnosis of synchronous or metachronous double or multiple cancers; (ii) contraindications to ED (anaphylaxis or hypoglycemia); (iii) active infection, or uncontrolled hypertension or diabetes; (iv) clinically relevant cardiac or pulmonary disease; (v) history of clinically relevant mental disorder or central nervous system disorder; (vi) pregnancy, intention for pregnancy, or lactation; (vii) participation in other clinical studies with overlapping endpoints; and (viii) unsuitability for the study as judged by the principal investigator.

### 2.2 Randomization in the original trial

All patients underwent gastrectomy with D1 and/or D2 lymphadenectomy between September 2011 and July 2012. Gastric reconstruction was carried out with the Billroth I method or Roux‐en‐Y anastomosis after DG, and with Roux‐en‐Y anastomosis after TG. Surgeons confirmed the eligibility criteria after the surgery. Patients were randomly assigned to the ED group or to the control diet group according to surgical method (TG/DG), clinical stage (≤IA/>IA), and presurgical BMI (<18.5/≥18.5 kg/m^2^). Patients with pathological stage II or III disease were given adjuvant chemotherapy with S‐1. For these patients, adjuvant chemotherapy is usually started within 6 weeks after surgery and given for 1 year.

The ED group received 300 kcal ED (Elental; EA Pharma Co., Ltd, Tokyo, Japan ) plus their regular diet for 6‐8 weeks after surgery, beginning from the day the patient started intake of soft rice or an equivalent diet after surgery. Meanwhile, the control group received regular diet alone.

The protocol stipulated that ED should be discontinued after completion of the protocol treatment in the ED group. However, nutritional intervention after the protocol treatment period was allowed without limitation on the duration and type of oral nutritional supplement in both groups if deemed necessary. Primary endpoint of the original trial was percentage of BWL (%BWL) from the presurgical body weight to the weight at 6‐8 weeks after surgery.

### 2.3 Data collection

The %BWL from presurgical body weight to the weight at 6 months and 1 year after surgery and the changes in nutrition‐related blood parameters (serum albumin and total lymphocyte count) at 6 months and 1 year after surgery were evaluated. Data on the duration and dosage of S‐1 adjuvant chemotherapy and on prolonged intake of oral nutritional supplement (longer than the maximum time predetermined under the protocol) were collected. Postoperative late complications ≥grade 3 according to the Common Terminology Criteria for Adverse Events (CTCAE), recurrence, and survival were assessed from 6 to 8 weeks to 1 year after surgery.

### 2.4 Study endpoints

Primary endpoint of this additional study was %BWL between the patients’ presurgical body weight and the weight at 1 year after surgery according to operation type. Secondary endpoints included changes in nutrition‐related blood parameters (serum albumin level and total lymphocyte count) at 1 year after surgery.

### 2.5 Statistical analysis

All statistical analyses were carried out with JMP Pro version 13.1 (SAS Institute Japan, Tokyo, Japan) and R version 3.5.1 (The Comprehensive R Archive Network). Differences were considered significant at *P* < .05. Fisher's exact test for categorical variables and two‐sample *t*‐test for numerical variables were used to assess differences between the two groups, as appropriate. For evaluation of treatment effect‐adjusted time point, a linear mixed‐effect model was applied with treatment and time point as fixed effect and subjects as random effect. Multiple regression analysis was carried out to identify independent factors potentially associated with %BWL at 1 year after surgery. A backward stepwise method with Bayesian information criteria was used for variable selection.

Data analyses were carried out using the modified intention‐to‐treat set, which was defined as all randomized patients excluding those who withdrew consent after enrollment but before randomization, or those without follow up.

## 3 RESULTS

### 3.1 Patient characteristics

**Figure 1:**
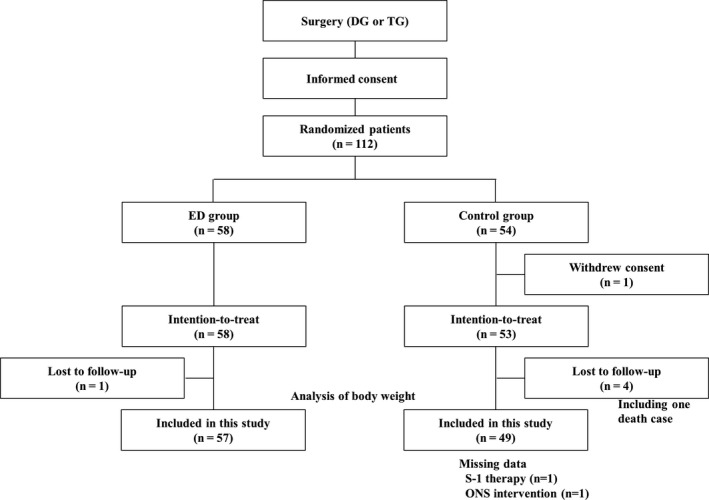
Study flow diagram. DG, distal gastrectomy; ED, elemental diet; ONS, oral nutritional supplementation; TG, total gastrectomy

Of the 112 patients who were registered in the original study, 106 patients were enrolled in this additional study because one patient withdrew consent and five patients were excluded owing to loss to follow up (Figure 1). Clinicopathological features of the patients are given in Table 1. There were 49 patients in the control group and 57 patients in the ED group. Of all patients, 31 received S‐1 adjuvant chemotherapy after surgery and eight were given oral nutritional supplement after completion of 6‐8 weeks of protocol treatment.

**Table 1:** Characteristics of the 106 analyzed patients at baseline and after surgery

		ED group (n = 57)	C group (n = 49)	*P*‐value
Age (years)	Average ± SD	66.6 ± 10.6	65.4 ± 11.4	.5558
Gender	Male	41	33	.6083
	Female	16	16	
Body weight (kg)	Average ± SD	57.5 ± 10.9	58.6 ± 12.5	.6488
Body mass index (kg/m^2^)	<22	30	24	.8685
	≧22	27	25	
Type of gastrectomy	TG	19	17	.8828
	DG	38	32	
Operative approach	Open	37	29	.5441
	Laparoscopic	20	20	
Reconstructive procedure	TG, Roux‐en‐Y	19	17	.9557
	DG, Roux‐en‐Y	13	10	
	DG, Billroth I	25	22	
Lymph node dissection	D1	6	5	.1685
	D1+	26	14	
	D2	25	30	
cStage	IA‐IB	35	28	.656
	IIA‐IV	22	21	
S‐1 adjuvant chemotherapy^a^	No	43	31	.3163
	Yes	14	17	
ONS at postoperative 1 year^b^	No	53	44	>.9999
	Yes	4	4	
Recurrence	No	51	44	.9567
	Yes	6	5	

Abbreviations: C, control; DG, distal gastrectomy; ED, elemental diet; ONS, oral nutritional supplementation; TG, total gastrectomy.

a Missing data of one case.

b Missing data of one case.

Two patients in the control group were transferred to other hospitals. Therefore, accurate information on S‐1 adjuvant chemotherapy and oral nutritional supplementation could not be obtained for these patients. There was a total of three adverse events of grade three or higher according to the CTCAE. Grade 3 abdominal pain (one patient) and grade 3 diarrhea (two patients) were observed only in the ED group. No patient underwent any type of surgery until 1 year after gastrectomy. Recurrence was observed in 11 patients. One patient died before 1 year after surgery. During the protocol treatment, mean ED amount was 11 287 ± 4120 mL in TG cases and 8581 ± 5270 mL in DG cases (*P* = .0547), and the median amount was 12 351 mL (3354‐16 560 mL) and 10 680 mL (0‐16 505 mL), respectively.

### 3.2 Nutritional intervention after protocol treatment

After the protocol treatment, oral nutritional supplement was taken by 11 patients (19.3%; TG: seven patients, DG: four patients) in the ED group and by six patients (12.5%; TG: six patients) in the control group (*P* = .5286). In the ED group, ED (Elental) was taken by nine patients and Ensure Liquid (ABBOTT JAPAN CO., LTD, Tokyo, Japan) was taken by two patients. Conversely, in the control group, ED was taken by three patients, Ensure Liquid by two patients, and RACOL‐NF Liquid (Otsuka Pharmaceutical Factory, Inc., Naruto, Japan) by one patient. Mean duration of additional oral nutritional supplementation after protocol treatment was 4.0 months in the ED group and 7.5 months in the control group (*P* = .0774), and median duration was 3.0 months (1.0‐10.0 months) and 10.0 months (1.5‐10.5 months), respectively. Four patients each in the ED and control groups received oral nutritional supplementation at postoperative 1 year. %BWL was significantly higher in patients with additional oral nutritional supplementation than in those without (12.9 ± 6.5% vs 7.3 ± 7.7%, *P* = .0062), and tended to be higher in patients who received oral nutritional supplementation at postoperative 1 year than in those who did not (12.2 ± 4.6% vs 7.9 ± 7.9%, *P* = .1332).

### 3.3 Long‐term outcomes

**Figure 2:**
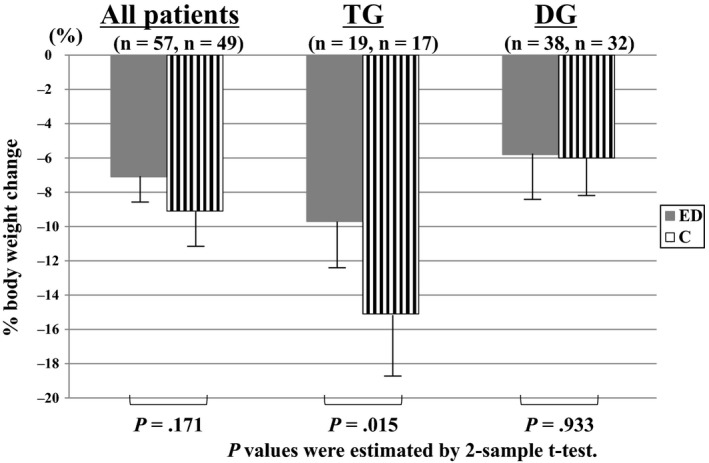
Percentage of body weight change between the patient's presurgical body weight and that at 1 year after surgery in all patients combined and in patients subdivided according to gastrectomy type (total or distal gastrectomy). C, control; DG, distal gastrectomy; ED, elemental diet; TG, total gastrectomy

**Figure 3:**
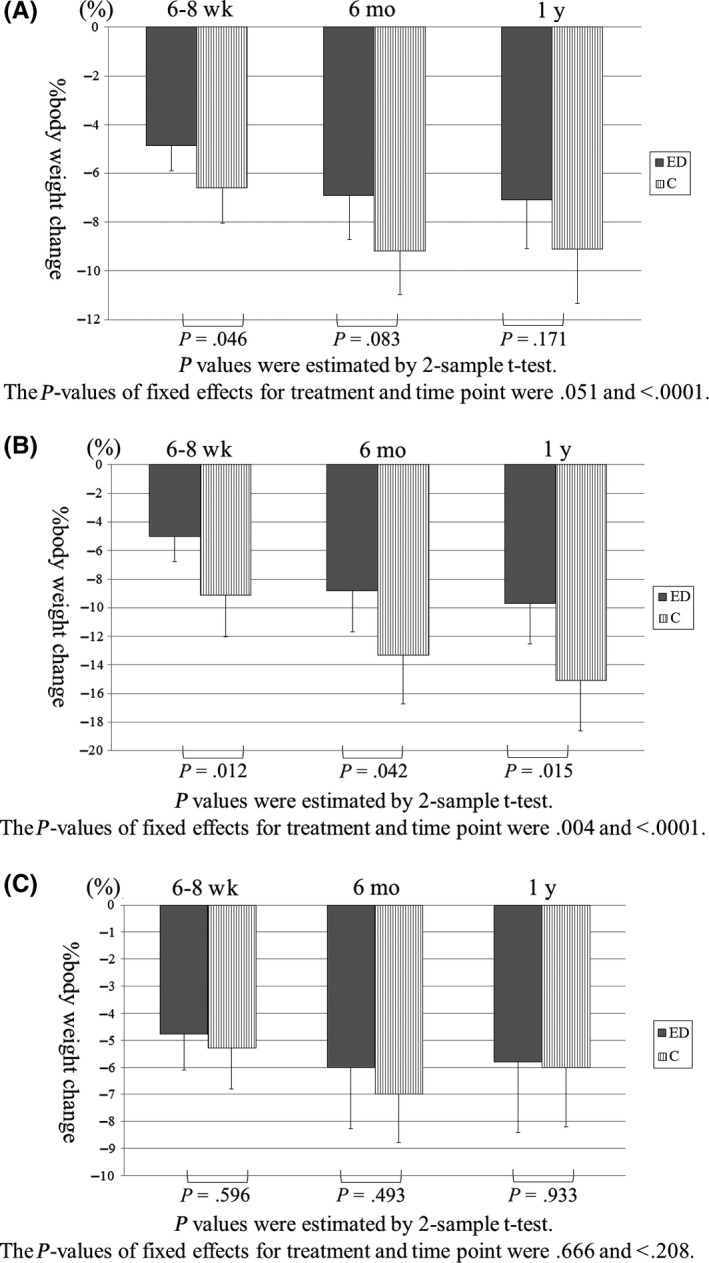
Percentage of body weight change after surgery in (A) all patients combined and in patients subdivided according to gastrectomy type (B: total or C: distal gastrectomy). C, control; ED, elemental diet

There was no significant difference in %BWL between the control group and the ED group (9.13 ± 7.72% [95% confidence interval, CI: 6.92‐11.35] vs 7.09 ± 7.49% [95% CI: 5.11‐9.08], *P* = .171) (Figure 2). In subgroup analyses, %BWL was significantly lower in the ED group than in the control group among patients who underwent TG (n = 19 and n = 17, respectively; 9.66 ± 5.98% [95% CI: 6.77‐12.54] vs 15.11 ± 6.78% [95% CI: 11.63‐18.60], *P* = .015), but not in those who underwent DG (n = 38 and n = 32; 5.81 ± 7.91% [95% CI: 3.21‐8.41] vs 5.96 ± 6.20% [95% CI: 3.72‐8.19], *P* = .933). At 6 months after surgery, no significant differences were found in all patients (Figure 3A), although %BWL in the ED group was smaller than that in the control group among patients who underwent TG (Figure 3B). There was no significant difference among patients who underwent DG (Figure 3C).

There were no significant differences between the control group and the ED group in the change in serum albumin level at 1 year after surgery regardless of surgical type; however, total lymphocyte count was higher in the ED group than in the control group at 1 year after surgery (2).

**Table 2:** Trends and rate of change in albumin level and in lymphocyte count at 1 year after surgery

	ED		C		*P*‐value
	Baseline	1 year	Baseline	1 year	
Serum albumin level (g/dL)					
Average ± SD	4.13 ± 0.40	4.14 ± 0.38	4.14 ± 0.41	4.08 ± 0.48	.735
Rate of change (%)	+0.50 ± 13.15		−0.38 ± 13.51		
Lymphocyte count (/μL)					
Average ± SD	1798 ± 511	1934 ± 634	1886 ± 804	1629 ± 618	.019
Rate of change (%)	+11.38 ± 37.24		−5.13 ± 35.13		

Abbreviations: C, control; ED, elemental diet.

### 3.4 Independent predictors of postoperative body weight loss

Multiple regression analysis showed that type of gastrectomy was independently associated with %BWL at 1 year after surgery. No association was found between the presence of S‐1 adjuvant chemotherapy or additional oral nutritional supplementation and %BWL (Table 3). Among the patients who underwent TG, ED was the only factor that affected BWL at 1 year after surgery (Table 4). In contrast, male gender was the only factor among the patients who underwent DG (Table 5).

**Table 3:** Multiple regression analysis and backward stepwise method to identify independent predictors of postoperative body weight loss

	n^a^	Multivariate analysis		Backward stepwise method	
		Coefficients	*P*‐value	Coefficients	*P*‐value
		[95% CI]		[95% CI]	
ED given (yes vs no)	57/47	−2.085 [−4.871, 0.701]	.141		
Type of gastrectomy (TG vs DG)	35/69	5.521 [2.452, 8.590]	.001	6.116 [3.199, 9.032]	<.001
Gender (male vs female)	72/32	2.427 [−0.688, 5.541]	.125		
Age (<65 vs ≥65 years)	46/58	−0.329 [−3.178, 2.520]	.819		
Body mass index (<22 vs ≥22 kg/m^2^)	53/51	−2.356 [−5.169, 0.458]	.1		
cStage (IA, IB vs II, III, IV)	62/42	−2.156 [−6.687, 2.375]	.347		
S‐1 adjuvant chemotherapy (yes vs no)	30/74	−0.868 [−5.831, 4.095]	.729		
ONS at postoperative 1 year (yes vs no)	7/97	0.178 [−5.818, 6.174]	.953		

Abbreviations: CI, confidence interval; DG, distal gastrectomy; ED, elemental diet; ONS, oral nutritional supplementation; TG, total gastrectomy.

Missing data of two cases.

**Table 4:** Multiple regression analysis and backward stepwise method to identify independent predictors of postoperative body weight loss in patients who underwent total gastrectomy

	n^a^	Multivariate analysis		Backward stepwise method	
		Coefficients	*P*‐value	Coefficients	*P*‐value
		[95% CI]		[95% CI]	
ED given (yes vs no)	19/16	−4.523 [−9.512, 0.466]	.074	−5.218 [−9.654, −0.781]	.023
Gender (male vs female)	26/9	0.55 [−5.574, 6.674]	.855		
Age (<65 vs ≥65 years)	16/19	3.404 [−1.820, 8.628]	.192		
Body mass index (<22 vs ≥22 kg/m^2^)	17/18	−0.475 [−5.605, 4.656]	.851		
cStage (IA, IB vs II, III, IV)	17/18	−1.389 [−8.433, 5.655]	.689		
S‐1 adjuvant chemotherapy (yes vs no)	12/23	−1.041 [−9.469, 7.387]	.802		
ONS at postoperative 1 year (yes vs no)	6/29	−0.405 [−8.242, 7.431]	.916		

Abbreviations: CI, confidence interval; DG, distal gastrectomy; ED, elemental diet; ONS, oral nutritional supplementation; TG, total gastrectomy.

a Missing data of one case.

**Table 5:** Multiple regression analysis and backward stepwise method to identify independent predictors of postoperative body weight loss in patients who underwent distal gastrectomy

	n^a^	Multivariate analysis		Backward stepwise method	
		Coefficients	*P*‐value	Coefficients	*P*‐value
		[95% CI]		[95% CI]	
ED given (yes vs no)	38/31	−1.223 [−4.718, 2.272]	.487		
Gender (male vs female)	46/23	3.186 [−0.683, 7.054]	.105	3.925 [0.355, 7.495]	.032
Age (<65 vs ≥65 years)	30/39	−2.02 [−5.560, 1.521]	.259		
Body mass index (<22 vs ≥22 kg/m^2^)	36/33	−2.441 [−6.046, 1.165]	.181		
cStage (IA, IB vs II, III, IV)	45/24	−3.081 [−9.310, 3.148]	.327		
S‐1 adjuvant chemotherapy (yes vs no)	18/51	−2.126 [−8.908, 4.656]	.533		
ONS at postoperative 1 year (yes vs no)	1/68	3.86 [−10.694, 18.413]	.598		

Abbreviations: CI, confidence interval; DG, distal gastrectomy; ED, elemental diet; ONS, oral nutritional supplementation; TG, total gastrectomy.

Missing data of one case.

## 4 DISCUSSION

This is the first study to show that short‐term nutritional intervention with ED at the early stage after TG has a positive effect on suppression of BWL not only in the short term after surgery but also after 1 year in prospectively registered and randomized cases. However, nutritional intervention with ED did not contribute at all to suppression of BWL after DG.

Body weight loss after gastrectomy is a problem concerning many surgeons. Many types of reconstruction methods and perioperative management after gastrectomy have been attempted, such as the enhanced recovery after surgery (ERAS) protocol.^13^, ^14^ As remarkable BWL occurs especially after TG, various reconstruction methods have been applied, such as jejunal pouch, jejunal interposition, or double‐tract reconstruction. However, no prospective trials have achieved striking improvement with respect to BWL.^15^, ^16^, ^17^, ^18^ Meanwhile, the ERAS protocol, which encompasses all types of perioperative management, has been recommended in gastric surgery for improving nitrogen balance under the condition of hypercatabolism associated with inflammatory reactions as a result of surgical stress, by shortening the fasting period as much as possible. Although ERAS is a reasonable management protocol, there remains a problem in that patients who undergo gastrectomy, in particular TG, are not able to consume a sufficient amount of food early after surgery because of loss of reservoir function and reduction in blood ghrelin levels.^19^, ^20^

According to many retrospective studies on body weight after gastrectomy, BWL occurs within approximately 1 month. Then, gradual weight loss continues by 3 or 6 months after surgery, and only slight weight reductions occur after 6 months regardless of the surgical procedure.^7^, ^8^, ^21^ In the present study, both in patients who underwent DG and in those who underwent TG, BWL occurred by 6 months and was almost stabilized after 6 months, consistent with previous studies. In patients who underwent DG, there was little difference in BWL between the control group and the ED group at 6 months and at 1 year, similar to that at 6‐8 weeks after surgery. In patients who underwent TG, there was an approximate 4% disparity in the early postoperative period between the control group and the ED group, and the difference in BWL between the groups was maintained until 1 year after surgery.

In the present study, there was a concern that additional oral nutritional supplementation after the protocol treatment might affect long‐term body weight. However, on the contrary, BWL was greater in patients with additional supplementation than in those without. This finding was believed to be attributable to the fact that oral nutritional supplement was given to patients who needed nutritional intervention because of reduced food intake and BWL associated with post‐gastrectomy syndrome in both groups. Therefore, additional oral nutritional supplementation would not have a critical impact on the results of this study.

In their retrospective study, Ohkura et al reported that early intervention with ED after DG, but not TG, reduced weight loss over the long‐term period (1, 3, 6 and 9 months) after surgery.^22^ Our result implies that short‐term intervention with ED for 6‐8 weeks from the early postoperative days contributes to suppression of BWL at 6‐12 months after surgery. Kobayashi et al showed that oral nutritional supplementation at >200 kcal/day led to a significant reduction in BWL at 3 months after surgery in gastrectomized patients.^23^ It would be interesting to see the long‐term difference between patients with a high rate of BWL and those with a low rate of BWL in the early postoperative period in prospective studies on oral nutritional intervention.

With respect to body composition after gastrectomy, skeletal muscle mass shows a sharp decline in the early days because of muscle catabolism and lack of food intake,^24^ and then slowly increases after the third month. Body fat decreases at almost the same pace by 6 months after surgery.^25^ It is important to suppress the reduction in skeletal muscle mass in addition to suppression of BWL after surgery because it was reported that loss of lean body mass might be an important risk factor for a decrease in compliance with adjuvant S‐1 chemotherapy.^26^, ^27^

Adherence to ED was better in patients who underwent TG than in those who underwent DG, as described previously.^12^ In TG cases with good adherence to ED, it is considered that replenishment of the protein source through ED from the early postoperative period may suppress decrease in body protein (i.e. decrease in skeletal muscle mass). In a rodent sarcopenia model induced by TG, giving branched‐chain amino acids and glutamine has been reported to be effective in suppressing BWL and skeletal muscle atrophy.^28^ Because ED contains branched‐chain amino acids and glutamine, the effects of ED may have been shown in patients in the TG group with low food intake but with good adherence to ED. It is presumed that if skeletal muscle reduction is suppressed early after surgery with ED intervention, the skeletal muscle activity after 2 months from surgery may be higher than that in the case of large skeletal muscle reduction early after surgery.

However, myokine, a cytokine secreted from skeletal muscle, has been found to be useful for regulation of whole‐body metabolism.^29^, ^30^, ^31^ Exercise can maintain or increase skeletal muscle mass, which, in turn, maintains myokine secretion, possibly leading to improved metabolism. Therefore, after the completion of the ED intervention, significant suppression of weight loss in the long term may be possible. However, to verify this finding, it is necessary to monitor detailed changes in body composition and myokine levels after surgery.

Sarcopenia is associated with worse prognosis in various diseases. Although BWL after gastrectomy has been reported to worsen the prognosis, there is still little evidence supporting this claim.^9^ However, it is possible that long‐term BWL after gastrectomy can lead to poor prognosis because BWL after gastrectomy is accompanied by at least skeletal muscle loss (one of the conditions of sarcopenia). In the present study, nutritional intervention suppressed long‐term BWL in TG cases; however, it is unclear whether it will improve the prognosis. Large‐scale randomized controlled trials are needed to determine the prognostic impact of early postoperative nutritional interventions.

This study has some limitations. First, no regard was given to dietary intake assessment during the period from operation day to 1 year after surgery. There was no significant difference in late complications related to digestive symptoms; however, it is unclear whether total dietary caloric intake or composition differed between the two groups. Second, this study analyzed patients who were enrolled prospectively and it was not conducted as an original trial but as an accompanying research. Owing to loss to follow up, we investigated only 90% of the patients who were registered in the original randomized trial, and total sample size was very small. Further clinical trials are needed to clarify optimal nutritional intervention in terms of nutritional supplement type, intervention duration, timing of the start of the intervention, and candidates for the intervention. Furthermore, future studies should show that postoperative nutritional intervention improves quality of life, adherence to S‐1 adjuvant chemotherapy, and long‐term prognosis of patients.

The present study showed that daily nutritional intervention with oral ED at 300 kcal/day for 6‐8 weeks in the early postoperative days reduced BWL not only at 6‐8 weeks but also at 1 year after surgery in patients who underwent TG.

## DISCLOSURE

Funding: This study was partly funded by EA Pharma Co., Ltd, Tokyo, Japan under contract, but was not involved in the planning, implementation, analysis, or publication of study results.

Conflicts of Interest: Authors declare no conflicts of interest for this article.

Approval of the Protocol: This research was approved by the institutional review board of each institution.

Author Contributions: *Study concept and design*: Y. Kimura and H. Imamura. *Provision of study materials and patients*: Y. Kimura, K. Nishikawa, K. Kishi, K. Inoue, J. Matsuyama, Y. Akamaru, S. Tamura, J. Kawada, T. Kawase, R. Kawabata, Y. Fujiwara, and H. Imamura. *Assembly of data and critical revision of the article*: H. Kanno and T. Yamada. *Statistical analysis of data*: T. Shimokawa. *Statistical analysis and interpretation of data*: Y. Kimura. *Final approval of the version to publish*: Y. Kimura. All authors approved the final version of the manuscript.
